# Supplementary figures and images for: Tumour Genome Characterization of a Rare Case of Pulmonary Enteric Adenocarcinoma and Prior Colon Adenocarcinoma
Source: J Pers Med. 2021 Aug 4;11(8):768. doi: 10.3390/jpm11080768 (PMC8398793; doi:10.3390/jpm11080768)

Supplementary Figure S1

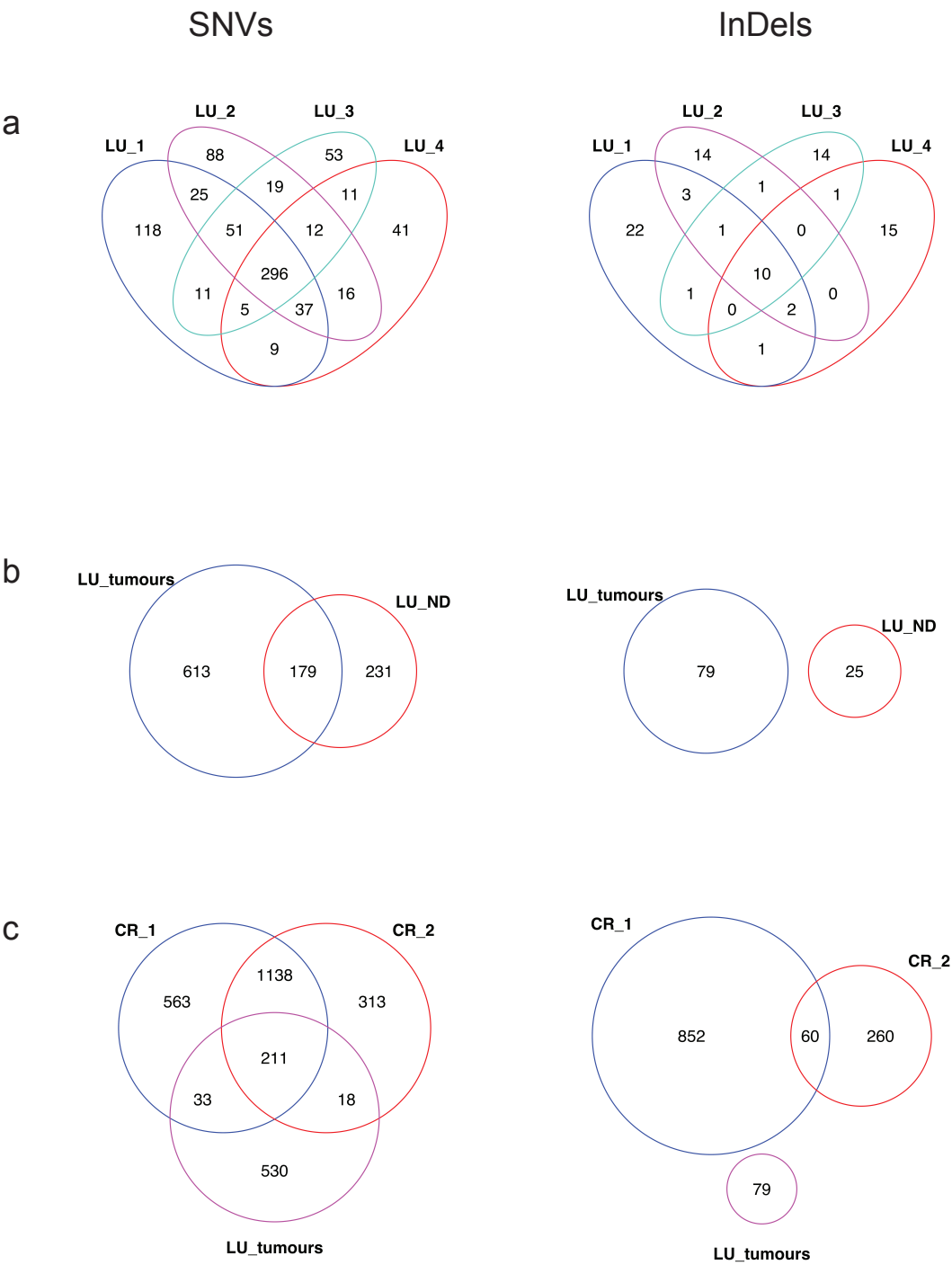

Supplement: Supplementary file 1 [file jpm-11-00768-s001.zip › jpm-1266375-supplementary.pdf]
